# EPIC: an evaluation of the psychological impact of early-phase clinical trials in cancer patients

**DOI:** 10.1016/j.esmoop.2022.100550

**Published:** 2022-08-19

**Authors:** P. Jittla, D.M. Graham, C. Zhou, J. Halliwell, S. O’Reilly, S. Aruketty, A. Azizi, T. Germetaki, J. Lowe, M. Little, G. Punnett, P. McMahon, L. Benson, L. Carter, M.G. Krebs, F.C. Thistlethwaite, E. Darlington, J. Yorke, N. Cook

**Affiliations:** 1 Experimental Cancer Medicine Team, The Christie NHS Foundation Trust, Manchester, UK; 2 Division of Cancer Sciences, Faculty of Biology, Medicine and Health, The University of Manchester, Manchester, UK; 3 CRUK Manchester Institute Cancer Biomarker Centre, University of Manchester, Manchester, UK; 4 Christie Patient Centred Research, The Christie NHS Foundation Trust, Manchester, UK; 5 Medical Oncology, Lancashire Teaching Hospitals NHS Foundation Trust, Preston, UK

**Keywords:** cancer, clinical trials, early phase, phase I, anxiety, depression

## Abstract

**Background:**

Anxiety and depression in patients with cancer is associated with decreased quality of life and increased morbidity and mortality. However, these are often overlooked and untreated. Early-phase clinical trials (EPCTs) recruit patients with advanced cancers who frequently lack future treatment options, which may lead to increased anxiety and depression. Despite this, EPCTs do not routinely consider psychological screening for patients.

**Patients and methods:**

This prospective observational study explored levels of anxiety and depression alongside impact of trial participation in the context of EPCTs. The Hospital Anxiety and Depression Scale and the Brief Illness Perceptions Questionnaire were completed at the point of EPCT consent, the end of screening and at pre-specified time points thereafter.

**Results:**

Sixty-four patients (median age 56 years; median Eastern Cooperative Oncology Group performance status 1) were recruited. At consent, 57 patients returned questionnaires; 39% reported clinically relevant levels of anxiety whilst 18% reported clinically relevant levels of depression. Sixty-three percent of patients experiencing psychological distress had never previously reported this. Males were more likely to be depressed (*P* = 0.037) and females were more likely to be anxious (*P* = 0.011). Changes in anxiety or depression were observed after trial enrolment on an individual level, but not significant on a population level.

**Conclusions:**

Patients on EPCTs are at an increased risk of anxiety and depression but may not seek relevant support. Sites offering EPCTs should consider including psychological screening to encourage a more holistic approach to cancer care and consider the sex of individuals when tailoring psychological support to meet specific needs.

## Highlights

• Early-phase cancer trial patients have an increased risk of anxiety and depression.
• Patients at risk were not seeking support for anxiety and depression.
• Male patients were more likely to be depressed; female patients were more likely to be anxious.
• This work highlights the need to screen for psychological symptoms in patients entering early-phase trials.

## Introduction

Anxiety and depression are not routinely assessed in cancer patients. Estimated prevalence is 10% (depression) and 20% (anxiety),^1^ though estimates vary depending on the screening method used. When using the Hospital Anxiety and Depression Scale (HADS),^2^ depression in cancer patients has been reported at ∼17%.^3^ These figures significantly exceed the prevalence of major depression in the general population, which is reported at 2%.^4^ In line with this, two-thirds of patients who have cancer and are experiencing depression will also have clinically significant anxiety symptoms,^5^ and symptoms of anxiety are more likely to coexist with depression than present as anxiety alone.^6^

The prevalence of anxiety and depression amongst cancer patients also varies depending on the treatment setting; lowest pre-treatment rising to highest following treatment.^7^ In addition to this, disease type and stage, younger age, lack of social support and poor prognosis are risk factors for depression and distress in cancer patients.^4^^,^^8^

In cancer patients, symptoms of depression and anxiety are strongly associated with decreased quality of life, diminished health status, poor treatment compliance and increased somatic symptom burden.^9^^,^^10^ The literature also demonstrates a strong association between depression and anxiety and increased morbidity and mortality in cancer patients,^11^, ^12^, ^13^, ^14^ emphasising the importance to address these symptoms. Early interventions within the palliative care setting have been shown to lead to significant improvements in quality of life, mood and survival in advanced cancer patients^15^ indicating the potential effectiveness of screening for symptoms early and using appropriate interventions.

Despite this, most psychological symptoms continue to be under-diagnosed and inadequately treated in cancer patients.^4^ Research shows that medical staff may underestimate the impact of psychological distress on cancer patients, and at times mistakenly assume that depression is a transient sadness and natural reaction to cancer.^16^ Yet when distress is recognised, this does not always translate into an appropriate referral for care or to a psychological service.^17^

Within oncology, experimental medicine and early-phase clinical trials (EPCTs) (defined as phase I and non-randomised phase II trials) typically include patients with advanced cancer, reduced life expectancy and limited treatment options, all of which are potential risk factors for anxiety and depression.^9^^,^^10^ However, most experimental cancer medicine trials do not consider psychological screening for patients.

There is limited literature investigating the levels of anxiety and depression in the EPCT population.^18^^,^^19^ This study aimed to evaluate the psychological impact of EPCTs on cancer patients and establish the levels of anxiety and depression within this population.

## Patients and methods

### Study design

This was a single-site, prospective, observational pilot study conducted within the Experimental Cancer Medicine Team (ECMT) at The Christie NHS Foundation Trust in Manchester, UK. The study was approved by the West Midlands–Solihull Research Ethics Committee on 14 August 2018 (18/WM/0209).

From September 2018 to June 2019, all patients referred to the ECMT for consideration of an EPCT were invited to participate. Inclusion criteria included ability to provide informed consent, ≥18 years of age and ability to comprehend English. Written informed consent for trial participation was obtained by research clinicians from patients who agreed to take part in the study.

### Patient-reported measures

Surrogate levels of anxiety and depression were self-assessed using the HADS (Appendix 1).^2^ This is a 14-item questionnaire, containing a 7-item subscale for both anxiety and depression in the previous week. Each item is on a scale of 0-3 and is loaded as appropriate to the question. Total scores for both subscales are calculated as follows:

• A score of 0-7 is considered normal.
• A score of 8-10 is considered a borderline case (borderline abnormal).
• A score of 11-21 is considered an abnormal case.

A score higher than 7 on either subscale (borderline case or abnormal case) is considered to be clinically significant.

The Brief Illness Perceptions Questionnaire (Brief-IPQ) (Appendix 2) was used to determine the illness perceptions in EPCT patients and rapidly assess the cognitive and emotional representation of their disease.^20^

### Data collection

Clinical and demographic information including disease status, medical history, age, ethnicity and sex was collected.

**Figure 1:**
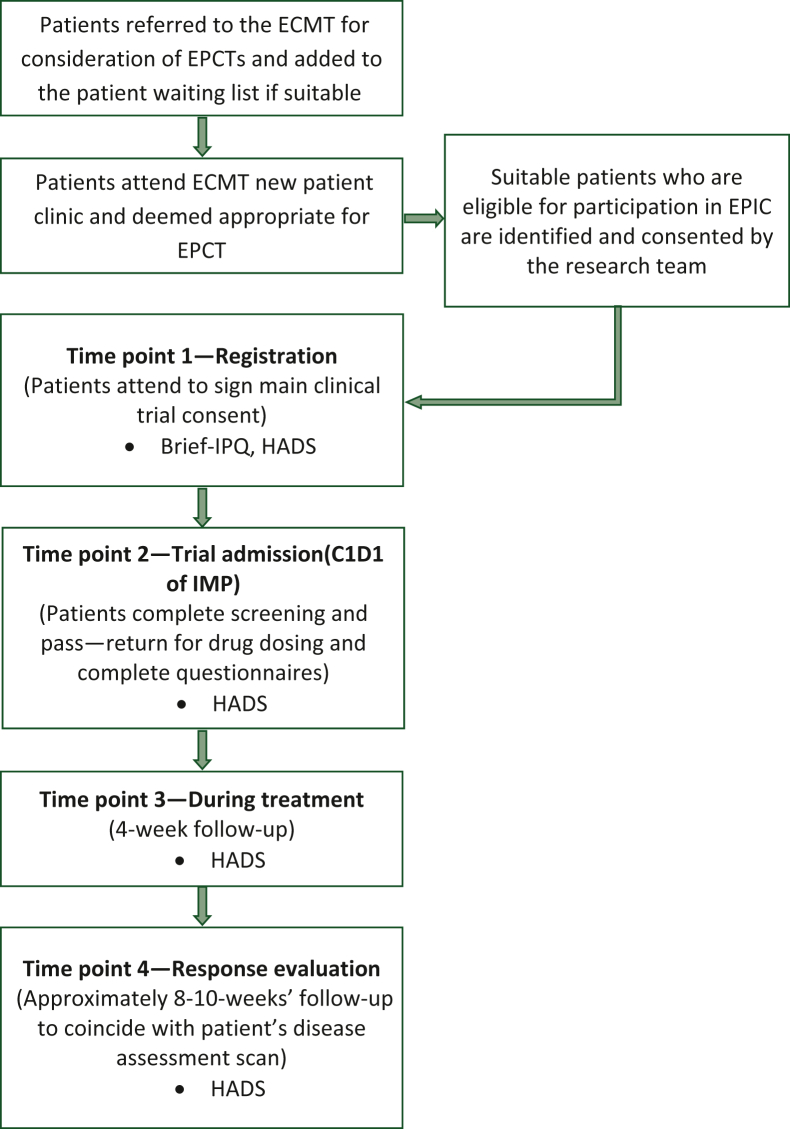
**Study schema flow diagram illustrating study design and derivation of patient sample.** Brief-IPQ, Brief Illness Perceptions Questionnaire; C1D1, cycle 1 day 1; ECMT, Experimental Cancer Medicine Team; EPCT, early-phase clinical trial; HADS, Hospital Anxiety and Depression Scale; IMP, investigational medicinal product.

The study schema is outlined in Figure 1. Participants who had no scheduled clinic visits within this period were able to receive and return questionnaires by mail. Where participants withdrew before the end of study, e.g. where they screen failed or discontinued treatment, data already collected were still used.

### Statistical analysis

Three different types of data were collected in this study: patient clinical data, self-assessed questionnaire data and anxiety and depression scores using the Brief-IPQ and HADS, respectively. The first contains both categorical and continuous variables, while the latter two are ordinal. The association between patient clinical data and depression/anxiety data at each time point was assessed using analysis of variance (ANOVA), with anxiety and depression scores log2 transformed and patient clinical data binarised by median if they were continuous variables. Similarly, the association between questionnaire data and anxiety and depression scores was assessed using ANOVA after binarising questionnaire data by median. A multivariable linear model was built based on each variable that is significantly associated with anxiety and depression scores.

The changes in anxiety and depression scores during treatment were assessed using two different ways: (i) the scores were compared at a population level using paired *t*-tests (after log2 transformation); (ii) score changes for each individual were calculated by their difference, and the association between the changes and the clinical and questionnaire data was calculated using ANOVA.

Treatment responses by RECIST v1.1^21^ were collected and the association with anxiety and depression score changes at an individual level was assessed using *t*-tests.

## Results

**Figure 2:**
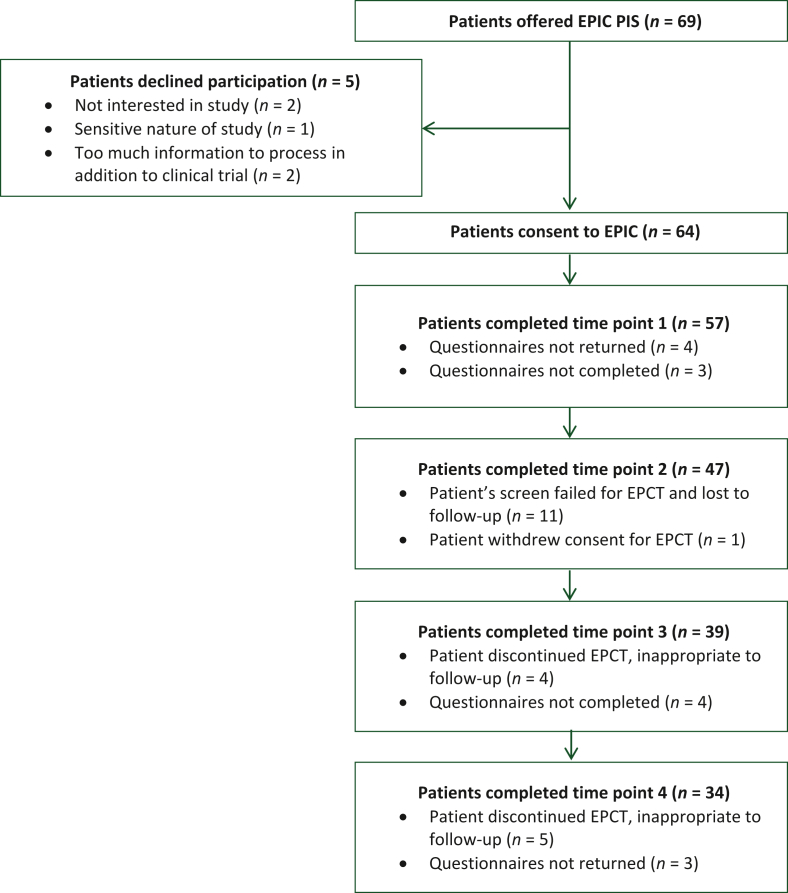
**CONSORT flow diagram of the dropout of patients as the study progresses**. EPCT, early-phase clinical trial; EPIC, An **E**valuation of the **P**sychological **I**mpact of Early Phase **C**linical Trials in Cancer Patients; PIS, patient information sheet.

Ninety-three percent (64/69) of participants offered the patient information sheet volunteered to take part and provided informed consent. Reasons for declining participation are noted in Figure 2. Participant attrition on study was notable [there was a 40% reduction (57 to 34) in the number of questionnaires completed between time points 1 and 4], primarily due to patients being ineligible for study and progression of disease which led to discontinuation from study. Participant characteristics are outlined in Table 1.

**Table 1:** Characteristics of study participants

Variable name	Value (%)
Age, years	
Median	58
Range	21-77
Sex	
Women	28 (44)
Men	36 (56)
Eastern Cooperative Oncology Group (ECOG) performance status	
0	18 (28)
1	44 (69)
2	2 (3)
Primary site of cancer	
Lung	15 (23)
Ovarian	10 (16)
Colorectal	10 (16)
Breast	8 (13)
Prostate	5 (8)
Other^a^	16 (25)
Previous lines of treatment	
0	2 (3)
1	8 (13)
2	18 (28)
≥3	36 (56)
Range	0-9
Median	3
Baseline symptoms	
0	19 (30)
1	15 (23)
2	15 (23)
≥3	15 (23)
Ethnicity	
White British	62 (97)
Asian	1 (1.5)
Mixed—White and Black African	1 (1.5)
Family history of cancer	
With family history	31 (48)
Without family history	25 (39)
Unknown	8 (13)
Occupational status	
Retired	26 (41)
Working	23 (36)
Not active	7 (11)
Unknown	8 (13)
Religious affiliations	
Christian	28 (44)
Agnostic	2 (3)
Atheist	2 (3)
None	32 (50)
Smoking history	
Never	34 (53)
Ex-smoker	18 (28)
Current	5 (8)
Unknown	7 (11)
Living arrangements	
With others	50 (78)
Alone	8 (13)
Unknown	6 (9)
Perceived levels of support	
Well supported	34 (53)
Not supported	21 (33)
Unknown	9 (14)

a Other primary sites included melanoma, anal, thymic, squamous cell carcinoma, solitary fibrous tumour, pancreatic, oropharynx, gastro-oesophageal, desmoid, chordoma, cholangiocarcinoma, bladder, ampullary and adenoid cystic.

**Figure 3:**
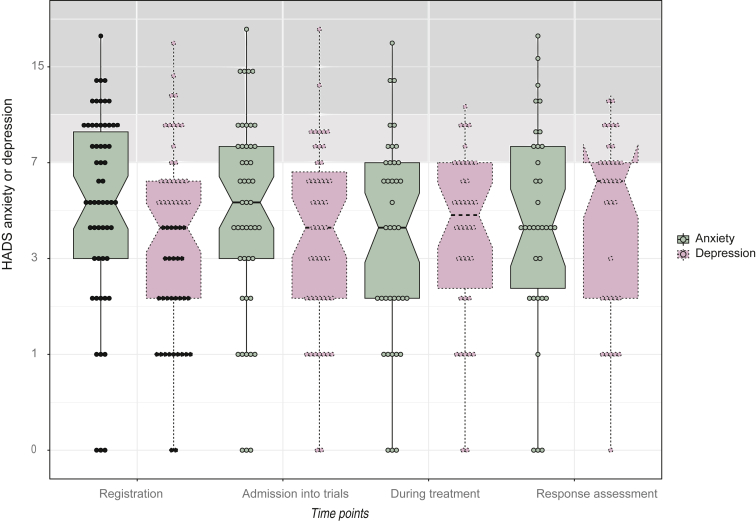
**Boxplot showing all anxiety and depression scores throughout the duration of the study.** HADS, Hospital Anxiety and Depression Scale.

At baseline, 39% of patients reported clinically significant anxiety scores. Anxiety showed a continued reduction from time point 1 (registration) to time point 3 (during treatment), but no clear trend was identified at a population level and the reduction was not found to be statistically significant at any time point (Figure 3).

**Figure 4:**
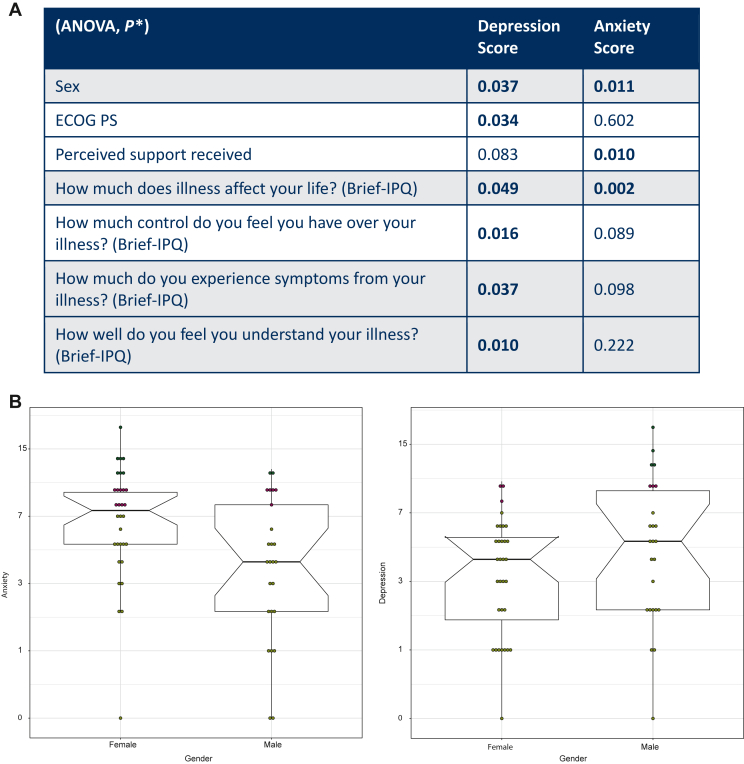
**Statistical analysis of clinical data.** (A) Statistically significant ANOVA results of clinical data and Brief-IPQ. ANOVA, analysis of variance; Brief-IPQ, Brief Illness Perceptions Questionnaire; ECOG PS, Eastern Cooperative Oncology Group performance status. ∗*P* < 0.05, significant. (B) Boxplots depicting significant trend with sex differences in relation to anxiety (more likely in females, *P* = 0.011) and depression (more likely in males, *P* = 0.037).

On an individual level, patients experiencing more symptoms at baseline were significantly more likely to have reduction in anxiety scores following enrolment on to the EPCT. Significantly less anxiety was also observed in those patients who had higher levels of perceived support (*P* = 0.010) (Figure 4A). At time point 4 (response assessment) when patients received their treatment response evaluation, both anxiety and depression levels increased but were not significant. However, the total HADS score did demonstrate significant elevation (*P* = 0.044).

Eighteen percent of patients reported clinically significant scores for depression at baseline. No statistically significant changes in depression were detected on a population level throughout the study. Patient’s Eastern Cooperative Oncology Group performance status (ECOG PS), which describes a patient’s level of functioning and ability to carry out daily tasks, was significantly associated with depression (*P* = 0.034) (Figure 4A), with a higher depression score observed in patients with a higher ECOG PS. Patients who self-reported a good understanding of their illness were also more likely to score higher for depression (*P* = 0.010).

Depression and anxiety scores were positively correlated (*r*^2^ = 0.495). Of the patients experiencing clinically relevant levels of anxiety and/or depression, 63% of these had never previously reported this to a health care professional. There was a significant trend with regard to sex; females presented as significantly more anxious (*P* = 0.011), whereas males presented as significantly more depressed (*P* = 0.037) (Figure 4B).

## Discussion

This study aimed to establish the levels of anxiety and depression in EPCT patients, determine illness perceptions in relation to anxiety and depression and characterise the psychological impact of EPCTs in cancer patients. We discovered that there is a significant psychological burden in EPCT patients which appears to be differentiated by sex. No statistically significant changes in the levels of anxiety or depression were observed before and after enrolment on to trial, suggesting that enrolling on to EPCTs alone has little impact on the psychological symptoms of cancer patients.

The work highlights the need to purposefully screen for psychological symptoms in EPCTs and supports the view that the low recognition of psychological distress can be improved by using simple validated screening tools.^22^ We found that the HADS is a suitable preliminary method of screening for psychological symptoms within a busy EPCT clinic, and this can easily inform which patients are most likely in need of further review and psychological support.

Our findings demonstrate substantial levels of potential anxiety and depression symptoms within this population, with 18% of patients reporting clinically relevant scores for depression symptoms and 39% of patients reporting clinically relevant scores for anxiety symptoms on the HADS at baseline. These findings are comparable to previously documented literature using the HADS in other cancer populations, as well as higher than the prevalence in the general population.^3^^,^^4^^,^^23^ It is possible that for EPCT patients, the thought of participating in a trial with many uncertainties, the considerations of starting a new treatment or recent confirmation of disease progression may all play some part in the levels of anxiety in this population. Regardless, adequate support should be provided for EPCT patients who are recognised to be at risk of anxiety and depression symptoms.

Of patients who were self-reporting clinically relevant scores of anxiety and depression on the HADS, 63% had never previously reported this to a health care professional. In line with previous research, many patients do not consider seeking help when experiencing psychological symptoms^4^ and it is likely that those most in need of treatment may be the least likely to access it. This suggests that a more active and systematic approach to screening for such symptoms is required. A proposed solution to this is to integrate validated tools such as the HADS questionnaire into the cancer patient pathway which requires no specialist staff training.

The findings also presented significant sex differences, with females more likely to present as anxious and males more likely to present as depressed. These findings are consistent with previous research in cancer patients^24^ but have not previously been noted within the EPCT population. Females tend to sustain healthier social networks, which may be protective against depression^25^ and often develop a better understanding of their illness over time, compared to males.^26^ Our results suggest that sex may be an important factor when tailoring psychological or educational interventions to the needs of EPCT patients.

A significant association was observed between ECOG PS and depression. Screening is essential in increasing symptom recognition and our data support the notion that ECOG PS may be used as a very inceptive screening tool for psychological symptoms.^27^ Similarly, patients who reported greater experience of symptoms from their illness reported higher depression scores. Within the cancer population, physical symptoms may lead to the development of depression^28^; however, depressive symptoms also have the potential to enhance the experience of physical symptoms, including pain and fatigue.^29^

Loss of control was significantly associated with depression. This finding is exacerbated further in patients who have a poor prognosis and can have a damaging psychological impact.^30^ Within the advanced cancer setting, patients often have concerns around a lack of control^31^ and the adoption of joint patient–clinician decision making with regard to treatment and trial options is important to restore this and empower patients. Importantly, good communication between patients and health care professionals, including the provision of educational resources, is crucial within the EPCT setting given the nature of more complex clinical trials and advanced disease.

This study has several limitations. A pragmatic approach to determine sample size was undertaken, due to time and resource restrictions. In addition, dropout was higher than expected; patients discontinuing on clinical trials and becoming too unwell to follow-up, as well as nine non-respondents via post, led to a steady decrease in sample size beyond baseline. Although the sample is small and from a single site, we are confident that this sample aligns phenotypically with the general EPCT population, given the median age, lines of treatment and ECOG PS scores. The use of standard structured clinical interviews to formally diagnose psychological symptoms may have provided a more accurate prevalence of formal anxiety and depression diagnoses in the population, compared to surrogate scores self-reported by patients on the HADS questionnaire.

Future research on a larger scale should determine prospectively whether the screening for psychological symptoms and subsequent evidence-based interventions improve the outcomes for EPCT patients, including quality of life and survival. With the number of patients on EPCTs increasing, as well as the adverse implications of the coronavirus disease-19 pandemic on the levels of anxiety and depression in the wider population, we believe this research is of timely importance.^32^

### Conclusion

This study established the prevalence of anxiety and depression on the EPCT patient population and is the first to our knowledge to evaluate the psychological impact of EPCTs in cancer patients. We have demonstrated the use of the HADS as an effective screening tool and potential surrogate for a formal diagnosis of depression or anxiety in the EPCT population. We strongly recommend the use of a screening tool for all EPCT patients, given their risk of anxiety and depression symptoms in order to address an avoidable cause of significant morbidity. Beyond this, the impact of provision of services such as implementation of psych-oncology referral pathways or direction to support tools should be formally assessed through clinical trials. The findings of the study have important implications for the way patients are assessed in the context of EPCTs and hope to inspire a more holistic approach towards experimental cancer medicine.

## References

[1] Mitchell A.J., Chan M., Bhatti H. (2011). Prevalence of depression, anxiety, and adjustment disorder in oncological, haematological, and palliative-care settings: a meta-analysis of 94 interview-based studies. Lancet Oncol.

[2] Zigmond A.S., Snaith R.P. (1983). The Hospital Anxiety and Depression Scale. Acta Psychiatr Scand.

[3] Krebber A.M.H., Buffart L.M., Kleijn G. (2014). Prevalence of depression in cancer patients: a meta-analysis of diagnostic interviews and self-report instruments. Psychooncology.

[4] Walker J., Hansen C.H., Martin P. (2014). Prevalence, associations, and adequacy of treatment of major depression in patients with cancer: a cross-sectional analysis of routinely collected clinical data. Lancet Psychiatry.

[5] Smith H.R. (2015). Depression in cancer patients: pathogenesis, implications and treatment (review). Oncol Lett.

[6] Die Trill M. (2013). Anxiety and sleep disorders in cancer patients. Eur J Cancer Suppl.

[7] Watts S., Prescott P., Mason J., McLeod N., Lewith G. (2015). Depression and anxiety in ovarian cancer: a systematic review and meta-analysis of prevalence rates. BMJ Open.

[8] Vodermaier A., Linden W., MacKenzie R., Greig D., Marshall C. (2011). Disease stage predicts post-diagnosis anxiety and depression only in some types of cancer. Br J Cancer.

[9] Brown L.F., Kroenke K., Theobald D.E., Wu J., Tu W. (2010). The association of depression and anxiety with health-related quality of life in cancer patients with depression and/or pain. Psychooncology.

[10] Colleoni M., Mandala M., Peruzzotti G., Robertson C., Bredart A., Goldhirsch A. (2000). Depression and degree of acceptance of adjuvant cytotoxic drugs. Lancet.

[11] Kroenke C.H., Kubzansky L.D., Schernhammer E.S., Holmes M.D., Kawachi I. (2006). Social networks, social support, and survival after breast cancer diagnosis. J Clin Oncol.

[12] Batty G.D., Russ T.C., Stamatakis E., Kivimäki M. (2017). Psychological distress in relation to site specific cancer mortality: pooling of unpublished data from 16 prospective cohort studies. BMJ.

[13] Lloyd-Williams M., Shiels C., Taylor F., Dennis M. (2009). Depression — an independent predictor of early death in patients with advanced cancer. J Affect Disord.

[14] Giese-Davis J., Collie K., Rancourt K.M.S., Neri E., Kraemer H.C., Spiegel D. (2011). Decrease in depression symptoms is associated with longer survival in patients with metastatic breast cancer: a secondary analysis. J Clin Oncol.

[15] Temel J.S., Greer J.A., Muzikansky A. (2010). Early palliative care for patients with metastatic non-small-cell lung cancer. N Engl J Med.

[16] Merckaert I., Libert Y., Delvaux N. (2005). Factors that influence physicians’ detection of distress in patients with cancer. Cancer.

[17] Keller M., Sommerfeldt S., Fischer C. (2004). Recognition of distress and psychiatric morbidity in cancer patients: a multi-method approach. Ann Oncol.

[18] Hunt A., Handorf E., Blau M. (2021). Psychological distress in patients with metastatic cancer enrolling on phase I clinical trials. J Cancer Surviv.

[19] Gad K.T., Lassen U., Duun-Henriksen A.K. (2021). Patients in phase 1 cancer trials: psychological distress and understanding of trial information. Acta Oncol.

[20] Broadbent E., Petrie K.J., Main J., Weinman J. (2006). The brief illness perception questionnaire. J Psychosom Res.

[21] Eisenhauer E.A., Therasse P., Bogaerts J. (2009). New response evaluation criteria in solid tumours: revised RECIST guideline (version 1.1). Eur J Cancer.

[22] Mitchell A.J., Meader N., Symonds P. (2010). Diagnostic validity of the Hospital Anxiety and Depression Scale (HADS) in cancer and palliative settings: a meta-analysis. J Affect Disord.

[23] Halliwell E., Main L., Richardson C. The fundamental facts: the latest facts and figures on mental health. 2007. https://www.mentalhealth.org.uk/explore-mental-health/publications/fundamental-facts-about-mental-health-2016.

[24] Linden W., Vodermaier A., MacKenzie R., Greig D. (2012). Anxiety and depression after cancer diagnosis: prevalence rates by cancer type, gender, and age. J Affect Disord.

[25] Curran E., Rosato M., Ferry F., Leavey G. (2020). Prevalence and factors associated with anxiety and depression in older adults: gender differences in psychosocial indicators. J Affect Disord.

[26] Fletcher K., Prigerson H.G., Paulk E. (2013). Gender differences in the evolution of illness understanding among patients with advanced cancer. J Support Oncol.

[27] Mei Hsien C.C., Wan Azman W.A., Md Yusof M., Ho G.F., Krupat E. (2012). Discrepancy in patient-rated and oncologist-rated performance status on depression and anxiety in cancer: a prospective study protocol. BMJ Open.

[28] Rodin G., Lo C., Mikulincer M., Donner A., Gagliese L., Zimmermann C. (2009). Pathways to distress: the multiple determinants of depression, hopelessness, and the desire for hastened death in metastatic cancer patients. Soc Sci Med.

[29] Fitzgerald P., Lo C., Li M., Gagliese L., Zimmermann C., Rodin G. (2015). The relationship between depression and physical symptom burden in advanced cancer. BMJ Support Palliat Care.

[30] Niedzwiedz C.L., Knifton L., Robb K.A., Katikireddi S.V., Smith D.J. (2019). Depression and anxiety among people living with and beyond cancer: a growing clinical and research priority. BMC Cancer.

[31] Volker D.L., Wu H.-L. (2011). Cancer patients’ preferences for control at the end of life. Qual Health Res.

[32] Duan L., Zhu G. (2020). Psychological interventions for people affected by the COVID-19 epidemic. Lancet Psychiatry.

