# Supplementary material for: EPIC: an evaluation of the psychological impact of early-phase clinical trials in cancer patients
Source: ESMO Open. 2022 Aug 19;7(5):100550. doi: 10.1016/j.esmoop.2022.100550 (PMC9420347; doi:10.1016/j.esmoop.2022.100550)

**Appendix 1**

HADS Questionnaire (Zigmond and Snaith, 1983)^2^


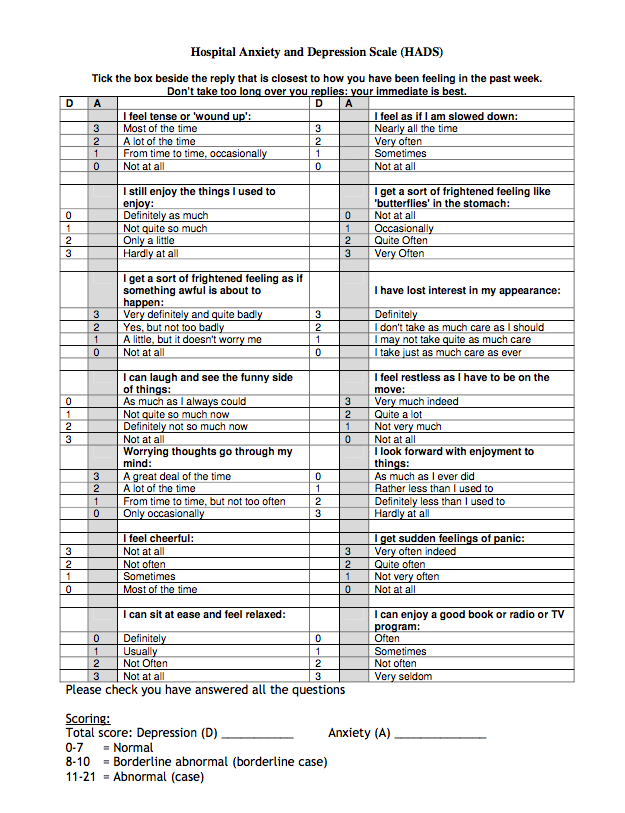


**Appendix2**

The Brief Illness Perception Questionnaire (Broadbent, Petrie, Main & Weinman, 2006)^20^


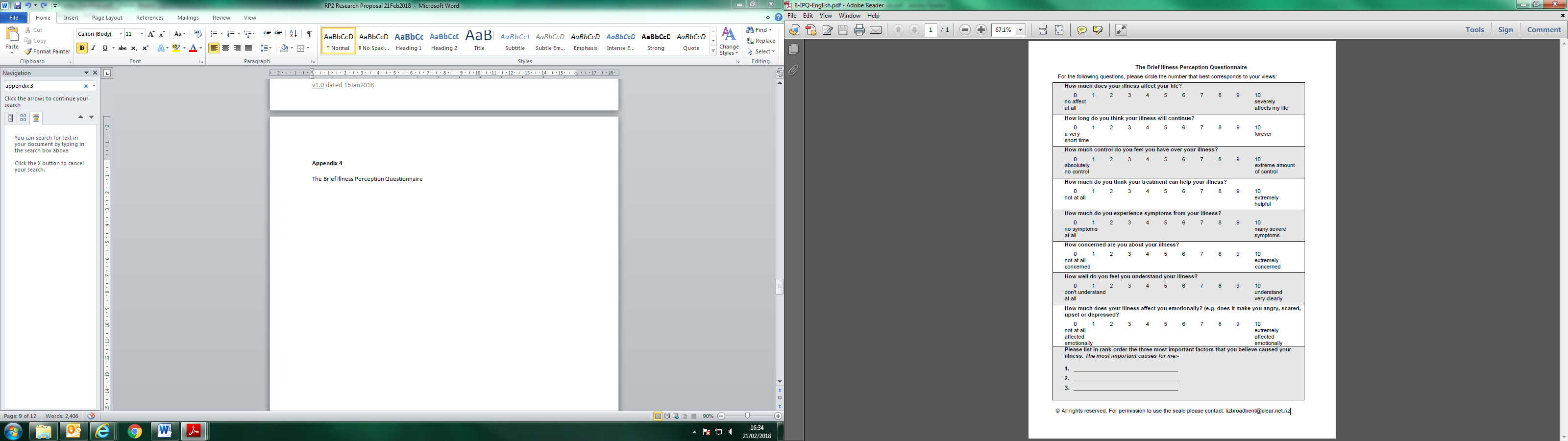

Supplement: Supplementary Material [file mmc1.docx]
